# The Untapped Potential of Ginsenosides and American Ginseng Berry in Promoting Mental Health via the Gut–Brain Axis

**DOI:** 10.3390/nu14122523

**Published:** 2022-06-17

**Authors:** Tristan St-Laurent, Riadh Hammami

**Affiliations:** 1 Department of Biochemistry, Microbiology and Immunology, Faculty of Medicine, University of Ottawa, Ottawa, ON K1H 8M5, Canada; tstla070@uottawa.ca; 2 School of Nutrition Sciences, Faculty of Health Sciences, University of Ottawa, Ottawa, ON K1N 6N5, Canada

**Keywords:** ginseng berry, ginsenosides, nutraceutical, gut microbiota, mental health, gut–brain axis

## Abstract

Despite the popularity of the ginseng (*Panax*) root in health research and on the market, the ginseng berry’s potential remains relatively unexplored. Implementing ginseng berry cultivations and designing berry-derived products could improve the accessibility to mental health-promoting nutraceuticals. Indeed, the berry could have a higher concentration of neuroprotective and antidepressant compounds than the root, which has already been the subject of research demonstrating its efficacy in the context of neuroprotection and mental health. In this review, data on the berry’s application in supporting mental health via the gut–brain axis is compiled and discussed.

## 1. Introduction

Mental illness is debilitating and compromises the individual’s quality of life, as well as it has surprisingly far-reaching economic effects, costing the Canadian economy an estimated 51 billion dollars annually [1]. Globally, mental illness has been estimated to be responsible for 32.4% of years lived with disability, surpassing all other forms of diseases [2]. Mood and anxiety disorders are the most common mental illnesses globally and in Canada [3], where their estimated prevalence is 4.7% annually [4]. Unfortunately, many standard pharmaceutical treatments, such as antidepressants, have significant side effects that could affect adherence to the treatment, as well as mixed results with regards to efficacy [5]. Furthermore, it has recently been shown that antidepressant use could negatively impact the intestinal microbiota diversity and be detrimental to certain types of beneficial bacteria [6,7]. With the concept and applications of the microbiota–gut–brain axis gaining traction among the scientific community, complementary treatments targeting this axis to promote mental health are needed.

Ginseng, one of the most important herbs of traditional Chinese medicine, has an impressive track record of positive effects in both in vitro and in vivo models of mental health [8], while also displaying efficacy in clinical research [9,10,11]. However, in traditional Chinese medicine and even in modern research, the root has been the primary focus of health allegations and, by extension, the focus of ginseng culture. The berries, which are largely regarded as by-products of the ginseng root culture, have great potential for applications in health due to their pharmacological properties and distinct composition with respect to the root [12]. Despite the rationale strongly supporting the pharmacological properties of the berries [12], they remain underutilized and are frequently discarded in agriculture, while the root is marketed. As ginseng is a slowly growing crop, root cultures take multiple years to harvest. Furthermore, significant crop mortality following the replanting of new ginseng is an issue plaguing agriculture; thus, bioremedial efforts have been undertaken to mitigate this effect [13]. The berry culture, on the other hand, presents numerous advantages. For instance, berries can be harvested from the same ginseng plant annually starting on the second year of growth, without any detriment to the crop. The root culture, in contrast, takes 4 to 10 years to achieve a minimal marketable maturity. Finally, the berry culture can be implemented without impacting the current root harvesting practices.

**Figure 1:**
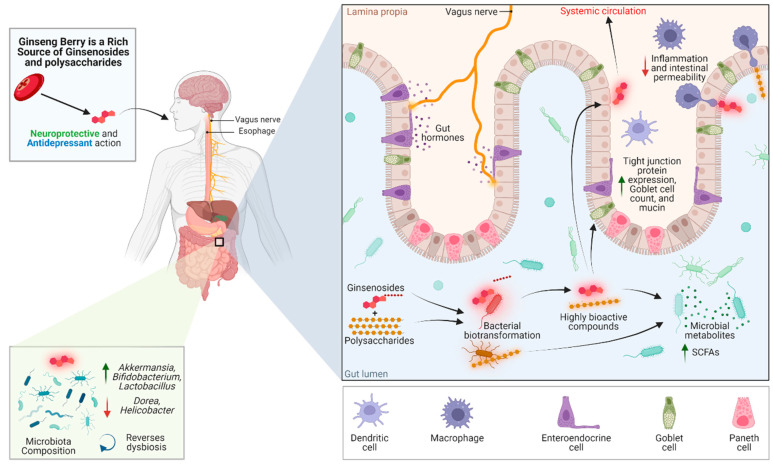
An overview of the reported studies highlighting the interplay of ginseng berry compounds with the microbiome–gut–brain axis. Green upward arrows represent a significant increase, whereas red downward arrows represent a significant decrease. Green arrow: increase; red arrow: decrease.

Here, we evaluate the potential of the ginseng berry as a promising source of bioactive compounds with mental health-promoting effects. This review also discusses the pharmacological mechanisms through the gut–brain axis in which ginseng could promote mental health, as shown in Figure 1.

## 2. The Berry Is a Highly Concentrated Source of Ginseng’s Therapeutic Compounds

The main bioactive compounds in the berry are of the same classes as those found in the root. Ginsenosides, usually denoted by a capital R followed by a lowercase letter and a number, if required (e.g., Rg1), are saponins present in an impressive diversity within the same type of ginseng and even in the same part of the plant. Their structural diversity is naturally accompanied by a diverse range of pharmacological functions and efficacy. Ginseng root polysaccharide extracts have also been researched in various contexts. As shown in Table 1, the berry could contain higher levels of neuroprotective and antidepressant bioactive compounds than the root.

It is reported that the total ginsenoside content could be higher in the berry than in the root by as much as 60% [12], though this is not consistent throughout the studies, perhaps due to varying harvest times and differences in the way that ginsenosides are measured in each study (e.g., measuring the root’s main ginsenosides but not the berry’s biases the total ginsenoside count). Rb3, Re, Rb2, Rd, and Rc, in descending order of abundance (with occasional variation between Re and Rb2), are the ginsenosides that are the most abundant in the American ginseng berry, and this is consistent throughout the studies assessing its composition via high-performance liquid chromatography [12,42,50,51]. Of note, the berry ginsenoside content can be different depending on the variety of ginseng selected. For instance, ginsenoside Re is the most abundant in Korean ginseng berries and is approximately 8 times more concentrated than Rb2 [52], whereas in the American ginseng, Re is at most 1.2 times as concentrated as Rb2 [12]. The harvest time has also been shown to cause significant variance in ginsenoside content; American ginseng berries were shown to lose over half of their Rb1, Re, and Rg1 content during the season, strongly suggesting that the ginsenoside content may be at its peak before the berries are ripe [18]. Post-harvest treatment should also be considered, as steaming has been shown to cause a sharp decrease in the total content, consistently causing a loss of about 50% after 2 h of steaming at 120 °C [42,50]. Conversely, ginsenosides Rh1, Rg2, (20)R-Rg2, Rg3, and Rh2 sharply increased in content after a 2 h steaming treatment [42,50].

Given the berry’s high concentration of Rb3, Re, Rb2, and Rd, the ginsenosides with demonstrated antidepressant and neuroprotective effects [20,22,27,30,31], it could be expected that the berry has even a superior potential for mental health applications than the root. Still, root extracts and specific ginsenosides have been the subject of most research and have consistently demonstrated efficacy in vitro and in vivo models in the context of central nervous system diseases and depression [53,54]. Another aspect to consider when evaluating the berry’s antidepressant and neuroprotective potential is that the microbial community of the intestine metabolizes the ginsenosides into alternate forms with varying effects and degrees of bioactivity. For instance, Rb3, the berry’s main ginsenoside, and its deglycosylated metabolites Rg3, Rh2, compound K, and 20(S)-protopanaxadiol have had their antidepressant potential assessed, and it was shown that Rg3 and compound K have more powerful antidepressant effects which are brought upon by the modulation of corticosterone, adrenocorticotropic hormone, and noradrenaline levels [55]. Thus, the fact that the berry has an inherently higher concentration of Rg3 than the root and a higher concentration of Rb3, which can, in turn, be deglycosylated into Rg3 [12,55], is a fine example of the berry’s untapped potential as a mental health-promoting nutraceutical.

## 3. Pharmacological Effects in The Context of Mental Health

Most of the mental health-promoting effects attributed to the berry come from extrapolation of data from single ginsenoside or total ginsenoside extract experiments. Data from experiments directly involving the berry or its distinct ginsenoside composition are scarce in the context of mental health. The berry saponin extract was shown to regulate 5-HT and rescue depressive-like behaviour in a mouse model of myocardial infarction, though this was fruit from the *Panax notoginseng* [56]. In fact, ginseng berry experiments with application to mental health seem to be limited to the examination of serotonin regulation in comorbid myocardial infarction models [57,58] and one additional study involving scopolamine-induced memory impairment, where the berry extract was shown to have antioxidant effects and to preserve acetylcholine and brain-derived neurotrophic factor (BDNF) mRNA levels [59]. This section illustrates the current mental health-related findings for the predominant ginsenosides in the American ginseng berry.

### 3.1. Ginsenoside Rb3

Rb3, the berry’s most abundant ginsenoside, exerts pharmacological effects that benefit mental health through multiple mechanisms. Such neuroprotective mechanisms occur through varied antioxidant effects, such as suppressing inducible nitric oxide synthase in hypoxic hippocampal neurons [60], preserving superoxide dismutase (SOD) and catalase (CAT) levels [61], and inducing Nrf2 transcription activity [62], which is downregulated in neurological conditions, such as depression [63]. Likewise, ginsenoside Rb3 was shown to interact with multiple neurotransmitters and receptors, leading to neuroprotective effects through inhibiting the NMDA receptor [64,65], activating the GABA(A) receptor [66], or acting beneficially on the noradrenergic pathway to relieve depression in rodent models [55,67].

### 3.2. Ginsenoside Re

Ginsenoside Re also demonstrates neuroprotective effects. It could be effective at reducing neuroinflammation by inhibiting the CAMK/MAPK/NF-κB signaling, as demonstrated by Madhi et al. [30], as well as by attenuating NLRP3 activation, as reported by Wang et al. [31]. In the same study, ginsenoside Re was also able to counter the loss of the antioxidant enzymes SOD, CAT, and glutathione (GSH) and the loss of Nrf2 expression following chronic restraint stress [31]. The compound also induced the expression of genes involved in acetylcholine neurotransmission, elevated acetylcholine levels, and enhanced the differentiation of Neuro-2a cells, which could translate to benefit in Alzheimer’s disease [68]. The neuronal effects also extend to reversing the depression- and anxiety-associated behavioural changes in rat models of repeated immobilization [32] and the learning and memory decline caused by chronic restraint in mice [31], while exerting BDNF-protecting effects in both studies.

### 3.3. Ginsenoside Rb2

Research evaluating the efficacy of ginsenoside Rb2 is scarce in the context of mental health, though it has been shown to protect against glutamate-mediated neurotoxicity in HT22 hippocampal cells [69]. Miao et al. have recently written a review compiling the pharmacological effects of Rb2, which include inhibition of oxidative stress, inflammation, and apoptosis through multiple pathways [20]. Although these effects were not tested in neurological models, some described pathways (SIRT1, AMPK, MAPK, and NF-κB) are relevant for many neurological conditions.

### 3.4. Ginsenoside Rd

Chen et al. have written a comprehensive review thoroughly describing the neuroprotective mechanisms of ginsenoside Rd, which was published a few months prior to this paper [27]. Some key reported data include anti-inflammatory effects via the regulation of iNOS, COX-2, MAPK, and NF-κB, antioxidant effects through increasing the SOD, GSH, and CAT, and antiapoptotic effects in several models of neuron stress [27]. Ginsenoside Rd was more recently shown to exert a significant antidepressant effect in the chronic unpredictable mild stress and behavioural despair mouse models via the hypoxia-inducible factor-1α and to increase the expression of SYN1 and PSD 95, two synaptic plasticity-related proteins [28]. Also of note, ginsenoside Rd alleviated both *Escherichia coli* K1-induced colitis and depression/anxiety in mice as measured by light/dark transition, forced swimming, and tail suspension tests, while significantly countering induced IL-6 expression in plasma and NF-κB activation (both colonic and hippocampal) [70]. In the same study, ginsenoside also protected the hippocampal BDNF levels and even reversed some changes in intestinal microbiota, brought upon by the administration of *Escherichia coli* K1 [70].

Ginsenosides could also exert neuroprotective effects through the modulation of microRNA, and Rd modulating miR-144-5p in a glioblastoma model is one such example [71]. In this study, Rd upregulated miR-144-5p, which decreased both TLR2 and the proliferation of the glioblastoma cells [71]. Although it remains to be confirmed that ginsenoside Rd could systemically upregulate miR-144-5p in vivo at a significant level, by extrapolating this microRNA’s targets to other models, it could be hypothesized that ginsenoside Rd has the potential to act therapeutically where TLR2 antagonism has shown benefit. For instance, anti-TLR2 has proven beneficial in decreasing α-synuclein accumulation in neuronal and astroglia cells in Parkinson’s and dementia with Lewy bodies mouse models, accompanied by decreased neuroinflammation and behavioural deficits [72]. Notably, miR-144-5p has been downregulated in depression and anxiety relative to healthy controls and inversely correlated with depression scores [73]. Similarly, a psychological treatment that decreased depression scores decreased specific inflammation-associated proteins and increased miR-144-5p in another cohort of depression, anxiety, and stress-related disorder patients [74]. Recently, Hyun compiled research demonstrating the microRNA modulating effects of various ginsenosides [75], but it may be too early to further extend these findings to the context of mental health. As microRNAs continue to gain traction as therapeutic targets, more research evaluating ginseng’s ability to modulate microRNAs would be of benefit to the scientific community. 

In summary, the American ginseng berry’s main ginsenosides are promising mental health-promoting compounds through multiple neuroprotective and anti-depressive mechanisms. As illustrated by a previously mentioned study involving *E. coli* K1 administration [70], the ginseng berry’s bioactive components can additionally exert mental health benefits by modulating microbiota and other intestinal health parameters.

## 4. The Ginseng Berry and The Gut–Brain Axis

Beyond direct pharmacological action on the nervous system, another mechanism through which ginseng could promote mental health is through the gut–brain axis. This axis, relating the concepts of the intestinal microbiome, intestinal barrier function, endocrine and neurological factors, and mental health, is of great importance as new implications for a wide range of disease states have been emerging. For instance, links have been established between the gut–brain axis and neurological conditions, such as Alzheimer’s disease, Parkinson’s disease, amyotrophic lateral sclerosis, stroke, and major depressive disorder [76,77], highlighting the need for more research evaluating strategies to target the gut–brain axis in these contexts effectively. Given the close link between host nutrition and the intestinal microbiome, nutritional and nutraceutical strategies are promising avenues to explore in helping treat these conditions. Potential therapeutic targets along the axis include the positive modulation of the intestinal microbiota composition or the reversal of dysbiosis, the reduced permeability of the intestinal epithelium to inflammatory food-derived antigens and inflammatory microbial products, and even the mitigation of the negative impact that psychotropic drugs could exert on the gut microbiome [78].

### 4.1. Intestinal Permeability

It is well known that with increased intestinal permeability, inflammatory microbial products, such as lipopolysaccharides (LPS), are present in higher quantities in the systemic circulation [79]. This endotoxemia results in metabolic dysfunction and neuroinflammation, potentially leading to overt depressive and anxious behaviour [80,81]. Indeed, serum LPS has been shown to dose-dependently depress mood in humans [81]. Further, the translocation of bacterial LPS into the systemic circulation is a major driver in the “leaky gut” model of depression [82]. There are currently insufficient data to determine the effect of the ginseng berry on the intestinal barrier function, though there is room for extrapolation. The effects of different ginseng extracts on relevant intestinal barrier function parameters, such as the tight junction proteins Claudin-1, Occludin, and Zonula Occludens-1 (ZO-1), colonic inflammatory markers, and serum markers of permeability, such as LPS and D-lactate, are reported in Table 2.

Overall, ginsenosides have remarkable potential as therapeutic products for preserving the intestinal barrier function in various stress situations through anti-inflammatory and transcriptional effects, favourably modulating tight junction protein expression. For instance, Seong et al.’s study involving a fermented ginseng root in a dextran sodium sulfate-induced murine colitis model has shown that the extract prevents the loss of the tight junction protein Zonula Occludens-1 while inhibiting the NF-κB inflammatory pathway [92]. The ginsenosides’ effects appear to go beyond mitigating the loss of tight junction proteins amidst inflammatory insult; ginsenosides have also been shown to upregulate the expression of tight junction protein expression and mRNA expression [97,99,100,101,104]. Ginseng has also been shown to increase *Muc2* expression [93], though it is unclear if this is a direct upregulation or due to the increase in goblet cell count that ginsenosides have also been shown to induce [96]. The effects discussed so far are complemented by improvements in histological parameters, such as the mucin barrier area or the thickness and reversal of epithelial damage from an impressive range of causes. Functional experiments of intestinal permeability have also demonstrated efficacy with decreases in serum LPS, replicated in a few different studies [99,101,104]. The ginseng root has also been shown to decrease serum beta-lactoglobulin following gastric administration in a mouse model of allergy [91] and decrease serum D-lactate and FITC-translocation across the intestinal epithelium in a rat model of intestinal injury by peritoneal air exposure [90]. However, research examining the impact of whole ginseng berries specifically on the intestinal barrier function is lacking. Furthermore, it is unknown whether berry polysaccharides or other non-saponin compounds could also exert a protective effect on the intestinal barrier, as root polysaccharides and oligopeptides have been shown to do.

### 4.2. Prebiotic Effects and Modulation of The Intestinal Microbiota

Another way the ginseng can act on the gut–brain axis is through modulation of the intestinal microbiome. This type of benefit to the axis contrasts with the previously discussed pharmacological effects, as they are indirect. Microbiome modulation is a multifaceted phenomenon whose results on the gut–brain axis depend on the initial host microbiome, diet, immunological factors, etc. Table 3 provides several examples of ginseng components reversing induced dysbiosis. However, it should be noted that ginseng also induces positive microbiome changes in healthy models, as well as improved intestinal metabolism and immunity, as shown by Sun et al. [105].

Ginseng and ginseng extracts have shown remarkable microbiome modulatory effects in an incredible amount of disease states and experimental diets. The reversal of deleterious microbiome changes is a persistent observation. For instance, the ginseng extracts have either increased or decreased the Firmicutes/Bacteroidetes ratio at the phylum level, whichever would reverse the changes brought upon by metabolic or intestinal dysfunction [94,97,101,106,113,116,117]. Based on the available literature, there appears to be a distinction between the effects of the root polysaccharides and the ginsenosides that can be made with regard to this ratio. Indeed, the ginsenosides appear to decrease the Firmicutes/Bacteroidetes ratio [94,97,101,106,117], whereas the root polysaccharides appear to increase it [113,116]. However, this distinction may be an oversimplification. It should be noted that the disease models in the experiments using ginsenosides were characterized by an increase in the Firmicutes/Bacteroidetes ratio, which the ginsenosides effectively reversed. In contrast, the models in the experiments using polysaccharides were characterized by a decrease in this ratio, which the polysaccharides also effectively reversed. It is unclear if the ginsenosides could also have acted to therapeutically increase the ratio if they had been used in a context where the increase would have acted as a reversal of dysbiosis, though it remains plausible. In supporting this idea, in vitro human microbiota-simulated fermentation with ginsenosides increased this ratio [124]. Together, these findings portend evidence that ginseng’s effects on the gut microbiome could be contextually adaptable to exert benefits. 

**Figure 2:**
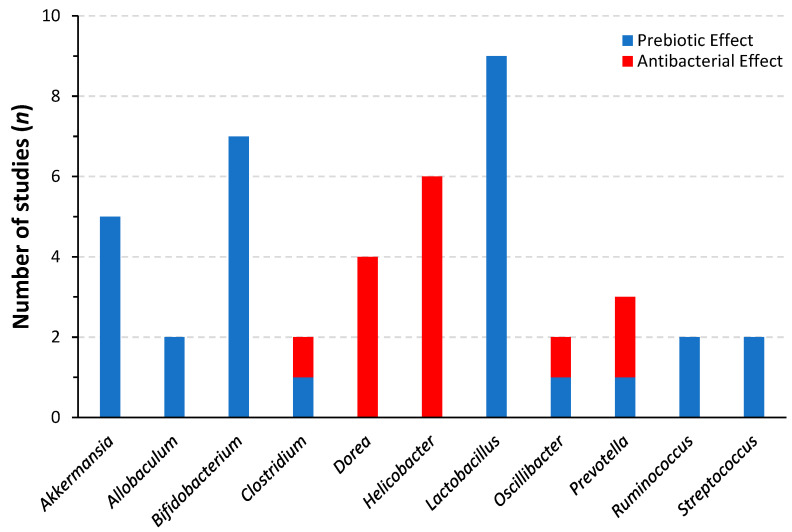
Reported prebiotic and antibacterial effects of ginseng and ginsenosides on microbial genera.

As shown in Figure 2, the ginseng extracts are relatively consistent in their effects on beneficial and detrimental bacteria from various genera. Indeed, ginseng’s prebiotic effect on beneficial bacteria of the genera *Akkermansia*, *Bifidobacterium,* and *Lactobacillus* is consistent throughout studies. The increase of *Akkermansia*, particularly *Akkermansia muciniphila*, is a beneficial characteristic associated with intestinal barrier functions [125,126]. The positive effects of *Bifidobacterium* and *Lactobacillus* are well known; these bacteria are associated with increased barrier function [127,128], which extends to metabolic benefits [129]. Of note, *Bifidobacterium* and *Lactobacillus* could have antidepressant effects [130,131] in their own right. Furthermore, consistent increases in short-chain fatty acid (SCFA) production following supplementation of extracted ginsenosides suggest that the saponins preferentially support the growth of SCFA-producing bacteria [98,100,101]. Increased production of luminal butyrate is of great benefit to the intestinal barrier function, as it has been shown to promote mucosal healing and production of protective mucus along the intestinal epithelium, and to the decrease in intestinal permeability by modulating epigenetic and transcriptional activity in the cells of the intestinal epithelium [132,133,134,135,136]. Another important piece of information to be extracted from this compilation of data is that despite ginseng’s prebiotic effects on beneficial bacteria, there is a homogenous observation that it exerts selective antibacterial effects on bacteria that are considered detrimental, such as those from the genera *Dorea* and *Helicobacter*. Of importance in this context is *Dorea*’s association with major depressive disorder [137]. 

### 4.3. Improved Health Functionality through Bioconversion

Most ginsenosides have inherently low bioavailability, therefore, ginseng bioconversion by the gut microbiome is critical for absorption by the host. Through microbial bioconversion processes, such as deglycosylation, the ginsenosides can achieve higher absorption rates and pharmacological activity [138,139]. Examples of metabolized ginsenosides include the most bioactive compound K and ginsenoside Rg3 [140]. Since this is a microbial process, the host’s microbiome significantly impacts the outcome of an administered dose of ginseng extract; thus, the bioconversion has varying effects between individuals [138]. Another aspect to consider in preparing extracts to improve health parameters is the apparent synergy between different plant components. For example, ginseng root polysaccharides have been shown to promote the microbial metabolism of co-administered ginsenoside Rb1 by a prebiotic effect in vivo while upregulating the intestinal uptake of Rb1 in vitro [114]. The observations of synergy have also been echoed in the context of efficacy for reversing cyclophosphamide-induced intestinal damage, where American ginseng ginsenosides and root polysaccharides were shown to have slightly different effects on inflammation, but were synergistic when co-administered [102]. To date, it remains unknown if berry polysaccharides and ginsenosides share the same synergistic relationship as root polysaccharides and ginsenosides. 

## 5. Safety

In 2021, a systematic review aiming to include all clinical trials involving all forms of ginseng was published [141]. Of the 121 retained studies that evaluated safety, 41.6% reported no adverse events, 31.6% reported no significant difference between groups in adverse events, and 26.6% reported no serious adverse events [141]. Mild adverse events included dizziness, headaches, diarrhea, insomnia, hypoglycemia, and nausea [141]. Due to the scarcity of human research involving the ginseng berry and its extracts, establishing clinical safety remains essential. A literature search yields two clinical studies involving the ginseng berry; the first is a 12-week study examining the efficacy and safety of a berry extract on a glycemic control [142]. The extract-treated group of 34 patients did not have any statistical difference in measured safety parameters except for a decrease in diastolic blood pressure compared to the placebo group of 38 [142]. There was also no statistically significant difference in the occurrence or type of adverse events in this study [142]. The second clinical trial, which lasted 8 weeks, similarly did not report any adverse events related to the use of the berries in the 59 volunteers, nor any changes in blood biochemistry and hormone and lipid panels relative to the placebo [143]. It should be noted that both studies used Korean ginseng berries. In summary, the safety can be extrapolated from clinical research involving different parts of the American ginseng (i.e., the root) or from clinical research using Korean ginseng berries. Both perspectives suggest a good safety profile, but confirmation of the American ginseng berry’s safety through human trials remains undone.

## 6. Conclusions

By examining the American ginseng berry’s saponin profile and extrapolating from ginseng extract research, it can be determined that it has promising potential as a mental health-promoting nutraceutical. Indeed, through its neuroprotective and antidepressant effects that are amplified through microbial bioconversion, its microbiota-modulating effects that reverse deleterious alterations in composition, as well as promote the growth of beneficial bacteria, and finally, its positive effects on intestinal epithelium inflammation and tight junction protein expression, ginseng could broadly impact the gut–brain axis. However, research evaluating the berry’s efficacy is in short supply and limited to preclinical studies. Some questions remain regarding the berry’s non-saponin compounds, such as polysaccharides and their bioactivity, as research is scarce in this area. Considering the berry’s numerous advantages at the agricultural level, as well as potential advantages in terms of ginsenoside composition, the implementation of the ginseng berry culture and the increase in research evaluating the berry’s clinical efficacy are strongly encouraged.

## Figures and Tables

**Table 1:** A review of American ginseng berry bioactive compounds—(*B>) content is significantly higher in the berry than root, (*B<) content is significantly less than the root, (B~) content is not statistically different from the root, (nil) was not detected in the berry, (ND) not determined in the study. Significance was determined using a two-tailed *t*-test with *p* < 0.05. When multiple harvest times were available in a study, the harvest date closest to August 30th was chosen.

Compounds		Pharmacological Effects	Content (Berry vs. Root, mg/g Dry Weight)
**Ginsenosides**	Rb1	Neuroprotective [14], anti-diabetic [15], mitochondrial antioxidant [16]	*B< [12,17] 0.86 ± 0.09 vs. 25.36 ± 1.67 [17] 9.03 ± 0.60 vs. ND [18] ND vs. 48.51 ± 1.79 [19] ND vs. 47.96 ± 1.04 [19]
	Rb2	Anti-diabetic, anti-viral, cardioprotective, neuroprotective [20]	*B> [12,17,21] 1.54 ± 0.95 vs. 0.3 ± 0.02 [17]
	Rb3	Anti-diabetic, anticonvulsant, antitumor, cardioprotective, antidepressant [22]	*B> [12,21]
	Rc	Antiallergic [23], antioxidant [24], anti-inflammatory [25], SIRT1 activation [26]	*B> [12] *B< [17] 1.51 ± 0.11 vs. 7.03 ± 2.15 [17]
	Rd	Neuroprotective, antioxidant, anti-inflammatory, neuroprotective [27], antidepressant [28]	B~ [12] *B< [17] 0.48 ± 0.1 vs. 3.16 ± 0.98 [17]
	Re	Cardioprotective [29], Neuroprotective [30,31], antidepressant [32]	*B> [12] *B< [17] 5.30 ± 0.54 vs. 17.45 ± 1.6 [17] 8.42 ± 0.19 vs. ND [18]
	Rg1	Stem cell regulation [33,34], anti-inflammatory [35], antidepressant [36]	*B< [12,17] 0.53 ± 0.09 vs. 2.39 ± 1.01 [17] 0.390 ± 0.010 vs. ND [18] ND vs. 3.15 ± 0.23 [19] ND vs. 2.49 ± 0.04 [19]
	Rg2	Cardioprotective [37,38,39,40], neuroprotective [41]	*B> [12]
	20(R)-Rg2	Insufficient data	nil [12,42]
	Rg3	Anticancer [43,44], neuroprotective [45]	*B> [12]
	Rh1	Anti-inflammatory, antioxidant, immunomodulatory, neuroprotective [46]	B~ [12]
	Rh2	Anti-cancer [47]	nil [12,42]
**Polysaccharides**		Anti-cancer [48,49]	

**Table 2:** Direct effects of ginseng on the intestinal barrier function.

Compounds	Models	Mechanism(s)	Significant Effects (*p* < 0.05)
American Ginseng Root Polysaccharides	Antibiotic-associated Diarrhea in Rats (Lincomycin Hydrochloride)	MAPK Signaling	Reduces colonic IL-1β, IL-6, IL-17A and TNF-α and increases IL-4 and IL-10. Increases Claudin-1 and Occludin expression [83]
Korean Ginseng Root Polysaccharides	DSS-induced Colitis in Rats	TLR4/MyD88/NF-κB-signaling pathway inhibition	Alleviates colitis symptoms, downregulates IL-1β, IL-2, IL-6, IL-17A, upregulates ZO-1 and Occludin [84]
Fermented Korean Ginseng Root Ginsenosides	Intraperitoneal LPS Injection in Mice	TLR4/MAPK	Attenuates LPS-induced increases in IL-6, TNF-α and IL-1β. Attenuates LPS-induced increases in ALT and AST, increases LPS-induced expression of Claudin-1 [85]
American Ginseng Ginsenosides	Cisplatin-induced intestinal injury in Mice	Decreased NF-κB activity	Attenuates cisplatin-induced increases in TNF-α and IL-1β. Attenuates cisplatin-induced decreases in ZO-1 and Occludin [86]
Korean Red Ginseng Root	MPTP-induced Intestinal Permeability in Mice	-	Prevents MPTP-induced decrease in Occludin and ZO-1, and MPTP-induced colonic increase in TNF-α and IL-1β [87]
Ginseng Polysaccharides (Unspecified Variety)	Intraperitoneal LPS Injection in Piglets	Decreased LPS-induced NF-κB activity	Increases jejunal villus height and expression of Occludin and Claudin in both LPS-treated and control groups. Alleviates LPS-induced increases in ALT, AST, TNF-α, and IL-1β [88]
Korean Ginseng Root Oligopeptides	Irradiation induced intestinal injury in mice	-	Decreases serum LPS levels and decreases plasma FITC-dextran. Pretreatment prevented plasma IL-6 decrease and TNF-α increase. Treatment dose-dependently increases ZO-1 and Occludin post-radiation injury [89]
Ginsenoside Rb1	Peritoneal air exposure intestinal damage in Rats	-	Dose-dependently reduces serum D-lactate and intestinal clearance of FITC-dextran [90]
Fermented and Unfermented Korean Red Ginseng Root	Ovalbumin-induced allergy in sensitized mice	Th1/Th2 balance, IgE suppression	Both treatments decrease IL-4 and TNF-α mRNA expression. Both treatments prevented an allergy-induced increase in serum beta-lactoglobulin after gastric administration [91]
Fermented Wild Ginseng Root	DSS-induced colitis Mouse Model	Decreased DSS-induced NF-κB activity	Alleviates colitis, prevents DSS-induced loss of ZO-1, downregulates DSS-induced IL-1β, IL-6, TNF-α, and IFN-γ mRNA expression. Decreases colonic levels of TNF-α [92]
Korean Ginseng	Healthy Mouse Model	-	Increased Muc2 expression [93]
Ginsenoside Rk3	High-fat diet Mouse Model	TLR4/NF-κB signaling pathway inhibition	Reduced colonic inflammatory cytokines and oxidative stress. Increases ZO-1, Occludin, and Claudin expression [94]
Ginsenoside Rh2	T-cell acute lymphoblastic leukemia mouse model	Decreased TLR4/MyD88 expression	Decreased IL-1β, IL-6, and TNF-α. Increased IL-10 and TGF-β. Increased mRNA expression of ZO-1, Claudin, and Occludin [95]
Ginsenosides Rb3 and Rd	ApcMin/+ mice (colon cancer model)	-	Increased Goblet and Paneth cell count [96]
Ginsenoside Rk3	DEN- and CCl4-induced Hepatocellular carcinoma mouse model	TLR4 pathway inhibition	Visual restoration of the intestinal barrier, increased expression of ZO-1, Occludin, and Claudin [97]
Ginsenoside Rk3	Lincomycin-treated mice	-	Increased expression of ZO-1, Occludin, and Claudin-1, and reversed structural changes to the epithelium. Prevented increased IL-1β, IL-6, IL-17, IFN- γ and TNF-α and prevented decreased IL-10 [98]
Ginsenoside Rg5	db/db diabetes mouse model	TLR4/NF-κB signaling pathway inhibition	Increased Occludin and ZO-1 protein expression, decreased serum LPS [99]
Panax Notoginseng saponins	Lepob mice on a high-fat diet	TLR4 pathway inhibition	Increased expression of ZO-1 and Claudin-1 [100]
Ginsenoside Rh4	Antibiotic intestinal inflammation mouse model	Decreased TLR4/NF-κB /MyD88 expression	Increased expression of ZO-1 and Claudin-1. Decreased IL-1β, IL-6, IL-17, IFN- γ and TNF-α. Prevented increase in IL-10. Reduced serum LPS [101]
American ginseng polysaccharides and ginsenosides	Cyclophosphamide-Induced Intestinal Damage in Mice	-	Both ginsenosides and polysaccharides independently increased mucin area, goblet cell count, and increased expression of ZO-1 and Occludin, but the combination had higher effect [102]
Ginsenoside Rg1	DSS-induced colitis mouse model	-	Decreased levels of IL-6, IL-33, TNF-α and increased IL-4 and IL-10 [103]
Korean Ginseng Ginsenosides	Mice on a high-fat diet	-	Increased expression of ZO-1 and Occludin mRNA expression. Decreased serum LPS [104]

MAPK = mitogen-activated protein kinase, IL = interleukin, ALT = alanine aminotransferase, AST = aspartate aminotransferase, TNF-α = tumour necrosis factor alpha, MPTP = 1-methyl-4-phenyl-1,2,3,6-tetrahydropyridine, FITC-dextran = fluorescein isothiocyanate-dextran, IFN- γ = interferon gamma.

**Table 3:** Direct effects of ginseng on the intestinal microbiome.

Compounds	Models	Significant Effects (*p* < 0.05)
American Ginseng Root Polysaccharides	Antibiotic-associated Diarrhea in Rats (Lincomycin Hydrochloride)	Increased production of acetate and propionate, improved the relative richness of *Lactobacillus* and Bacteroides, and reduced the relative richness of *Blautia* and *Coprococcus* [83]
Korean Ginseng	Healthy Mouse Model	Increased total bacterial count and *Lactobacillus* count [93]
Ginsenoside Rk3	High-fat diet Mouse Model	Increased abundance of *Bacteroides* and *Bifidobacteria*, decreased abundance of *Firmicutes* [94]
25-hydroxyl-protopanaxatriol	High-fat diet Mouse Model with streptozotocin	Partly reversed an increase in Firmicutes/Bacteroides ratio, increased relative abundance of Lachnospiraceae [106]
Fermented Wild Ginseng root	Antibiotic-associated diarrhea mouse model	Increased recovery of total bacteria counts after antibiotic treatment. Increased recovery of *Lactobacillus murinus*, *Bifidobacterium*, *Enterobacteriaceae bacterium,* and *Enterococcus faecium* [107]
Ginsenoside Rh2	T-cell acute lymphoblastic leukemia mouse model	Increased abundance of Bacteroides and Verrucomicrobia, decreased abundance of Firmicutes and Proteobacteria. Increased relative abundance of *Akkermansia*, *Lactobacillus,* and Lachnospiraceae [95]
Korean red ginseng root insoluble fiber	In vitro colon-simulated fermentation using swine fecal bacteria	Increased production of short-chain fatty acids, decreased alpha-diversity, and increased relative abundance of *Bifidobacterium* and *Prevotella* compared to control fermentation with cellulose [108]
Fermented Korean Ginseng Root	Alcoholic injury mice (ethanol diet)	Prevented relative abundance loss of *Akkermansia* and *Allobaculum*. Decreased relative abundance of *Parabacteroides* [109]
Ginseng Root Polysaccharides (Unspecified variety)	Healthy Piglets with supplemented diet	Increased colonic acetic acid, isobutyric acid, and butyrate. Decreased abundance of *Malainabacteria* [110]
Water Soluble Neutral Ginseng Polysaccharides	Antibiotic-associated Diarrhea in Mice (Lincomycin Hydrochloride)	Increased abundance of *Lactobacillus*, decreased abundance of *Bacteroides, Streptococcus, Ochrobactrum,* and *Pseudomonas* [111]
Unspecified Ginseng Extracts (Article in Chinese)	Healthy Rats	Increased abundance of *Bifidobacterium, Lactobacillus, Allobaculum,* and *Clostridium*. Decreased abundance of *Butyricimonas, Parabacteroides, Alistipes*, and *Helicobacter* [112]
Korean Red Ginseng Root Polysaccharides and Ginsenoside Rb1	Streptozotocin-Induced Diabetes Mouse Model	Polysaccharide treatment reversed the dysbiosis caused by the treatment, as evidenced by reversal of loss of relative abundance of Firmicutes and reversal of increase of the relative abundance of Bacteroides [113]
Ginseng Root Polysaccharides	DSS-induced Colitis Mouse Model	Reverses DSS-induced changes; increases abundance of *Bifidobacterium, Lactobacillus*, and the bacteria *Clostridium leptum* and *Clostridium coccoides*. Reduces abundance of Enterobacteriaceae and the bacterium Bacteroides fragilis [114]
Ginsenosides Rb3 and Rd	Apc^Min/+^ mice (colon cancer model)	Decreased abundance of *Dysgonomonas*, *Porphyromonas,* and *Parabacteroides*. Increased abundance of *Prevotella* and *Paraprevotella* (Rd only). Increased richness of family Bacteroidaceae; promoted growth of *Bacteroides vulgatus, Bacteroides xylanisolvens, Bacteroides gallinarum,* and *Bacteroides acidifiaciens* [96]
American Ginseng Root	AOM/DSS intestinal inflammation and tumorigenesis mouse model	Gradual reversal of loss of alpha-diversity and beta-diversity following DSS treatment. Reversed increase in *Bacteroidaceae*, *Porphyromonadaceae, Enterobacteriaceae,* and *Verrucomicrobiaceae*, and reversed the decrease in *Clostridiaceae, Catabacteriaceae*, *Lachnospiraceae, and Ruminococcaceae* [115]
Ginsenoside Rk3	DEN- and CCl4-induced Hepatocellular carcinoma mouse model	Reversed decrease in Bacteroidetes and increase in Firmicutes. Reversed decrease in Lachnospiraceae and Bifidobacteriaceae. Reversed increase in Ruminococcaceae. Reversed increase in *Helicobacter* and reversed the decrease in *Akkermansia*, *Lactobacillus*, *Oscillibacter*, and *Bifidobacterium* [97]
Korean Ginseng Root Polysaccharides	DSS-induced colitis in Mice	Restored loss of alpha diversity (Shannon Index). Reversed relative increase in *Bacteroidetes, Verrucomicrobia, Proteobacteria, Tenericutes, Cyanobacteria, Prevotella and Deferribacteres and reversed loss of Firmicutes and Akkermansia* [116]
Ginsenoside Rk3	Lincomycin-treated mice	Preserved Simpson, Shannon, ACE and Chao1 index at levels of control. Increased levels Bacillaceae, Bacteroidaceae and Prevotellaceae. Increased levels of Anaerostipes, Alloprevotella, Lachnoclostridium and Blautia. Decreased loss of acetic acid production, prevented decrease of propionic acid, butyric acid, isobutyric acid, and valeric acid production [98]
Ginseng Root Water-Soluble Extract (Unspecified Variety)	Exercise-Fatigue Mouse Model	Reversed relative loss of Bacteroidetes and reversed relative increase of Firmicutes. Increased Lactobacillus and Bacteroides, decreased Anaerotruncus. Reversed loss of Bifidobacterium, Streptococcus, Coprpcoccus, and Clostridium [117]
Protopanaxadiol-type Ginsenosides Extracted from Korean Ginseng Root	Human Fecal Microbiota In Vitro Fermentation	Increased relative abundance of *Escherichia-Shigella, decreased relative abundance of Dorea*, *Prevotella*, and *Megasphaera. Increased abundance of Lachnospiraceae, Streptococcaceae… (Abridged)* [118]
Korean Ginseng Root	Middle-Aged Korean Women with Obesity	Decreased relative abundance of *Anaerostipes* [119]
Korean Red Ginseng Root	Patients with non-alcoholic steatohepatitis	Increased *Lactobacillus* in subgroup who experienced improvements in ALT [120]
Ginsenoside Rg5	db/db diabetes mouse model	Reversed decrease in abundance of *Alloprevotella*, *Barnesiella*, *Coprobacter*, *Lactobacillus*, *Lactococcus*, and *Parasutterella*, reversed increase in abundance of *Oscillibacter*, *Clostridium*, *Helicobacter,* and *Dorea* (abridged) [99]
*Panax notoginseng* saponins	Diet-induced obesity mice	Increased abundance of *Akkermansia muciniphila* and *Parabacteroides distasonis* [121]
Ginsenoside Rb1	Diet-induced obesity mice	Decreased Helicobacteraceae and Ruminococcaceae, and enriched Rikenellaceae. Decreased abundance of *Dorea*, *Helicobacter* and *Oscillospira* [122]
*Panax Notoginseng* saponins	Lep^ob^ mice on High-fat diet	Increased fecal acetic acid, butyric acid, propionic acid, isobutyric acid, valeric acid and isovaleric acid [100]
Ginsenoside Rh4	Antibiotic intestinal inflammation mouse model	Decreased Firmicutes/Bacteroidetes ratio. Increased fecal acetic acid, butyric acid, propionic acid, isobutyric acid, valeric acid and isovaleric acid [101]
American ginseng polysaccharides and ginsenosides	Cyclophosphamide-Induced Intestinal Damage in Mice	The combination increased abundance of Clostridiales, *Bifidobacterium*, and Lachnospiraceae, and decreased abundance of Escherichia-Shigella and Peptococcaceae (reversing detrimental changes in microbiota). Polysaccharides and ginsenosides had different and synergistic effects [102]
Korean Ginseng polysaccharides and ginsenosides	Exhaustion by forced swimming and human hepatoma HepG2 cells xenograft	The combination reversed the changes in microbiota. Polysaccharides and ginsenosides had different and synergistic effects [123]
Ginsenosides	Human Fecal Microbiota In Vitro Fermentation	Increased relative abundance of Firmicutes and Proteobacteria and decreased relative abundance of Bacteroidetes. Increased abundance of *Escherichia*, *Streptococcus* and *Ruminococcus.* Decreased abundance of *Dorea*, *Sutterella*, *Prevotella* and *Megasphaera* [124]
Ginsenoside Rg1	DSS-induced colitis mouse model	Increased relative abundance of Lachnospiraceae and decrease of *Staphylococcus, Bacteroide* and *Ruminococcaceae* [103]
Korean Ginseng Ginsenosides	Mice on High-fat diet	Increased abundance of Parabacteroides, Muribaculaceae, Akkermansia, and Ruminococcus. Decreased abundance of Lachnospiraceae and *Helicobacter* [104]
Korean Ginseng	Healthy Rats	Increased abundance of *Bifidobacterium* and *Lactobacillus* [105]

## Data Availability

Not applicable.

## References

[1] Lim K.L., Jacobs P., Ohinmaa A., Schopflocher D., Dewa C.S. (2008). A New Population-Based Measure of the Economic Burden of Mental Illness in Canada. Chronic Dis. Can..

[2] Vigo D., Thornicroft G., Atun R. (2016). Estimating the True Global Burden of Mental Illness. Lancet Psychiatry.

[3] (2016). Public Health Agency of Canada Report from the Canadian Chronic Disease Surveillance System: Mood and Anxiety Disorders in Canada. https://www.canada.ca/en/public-health/services/publications/diseases-conditions/report-canadian-chronic-disease-surveillance-system-mood-anxiety-disorders-canada-2016.html.

[4] Lam R.W., McIntosh D., Wang J., Enns M.W., Kolivakis T., Michalak E.E., Sareen J., Song W.-Y., Kennedy S.H., MacQueen G.M. (2016). Canadian Network for Mood and Anxiety Treatments (CANMAT) 2016 Clinical Guidelines for the Management of Adults with Major Depressive Disorder. Can. J. Psychiatry.

[5] Almohammed O.A., Alsalem A.A., Almangour A.A., Alotaibi L.H., Al Yami M.S., Lai L. (2022). Antidepressants and Health-Related Quality of Life (HRQoL) for Patients with Depression: Analysis of the Medical Expenditure Panel Survey from the United States. PLoS ONE.

[6] Ait Chait Y., Mottawea W., Tompkins T.A., Hammami R. (2020). Unravelling the Antimicrobial Action of Antidepressants on Gut Commensal Microbes. Sci. Rep..

[7] Vich Vila A., Collij V., Sanna S., Sinha T., Imhann F., Bourgonje A.R., Mujagic Z., Jonkers D.M.A.E., Masclee A.A.M., Fu J. (2020). Impact of Commonly Used Drugs on the Composition and Metabolic Function of the Gut Microbiota. Nat. Commun..

[8] Kim Y., Cho S.-H. (2021). The Effect of Ginsenosides on Depression in Preclinical Studies: A Systematic Review and Meta-Analysis. J. Ginseng Res..

[9] Braz A.S., Morais L.C.S., Paula A.P., Diniz M.F.F.M., Almeida R.N. (2013). Effects of *Panax Ginseng* Extract in Patients with Fibromyalgia: A 12-Week, Randomized, Double-Blind, Placebo-Controlled Trial. Rev. Bras. Psiquiatr..

[10] Jeong H.-G., Ko Y.-H., Oh S.-Y., Han C., Kim T., Joe S.-H. (2015). Effect of Korean Red Ginseng as an Adjuvant Treatment for Women with Residual Symptoms of Major Depression. Asia-Pac. Psychiatry.

[11] Lee K.J., Ji G.E. (2014). The Effect of Fermented Red Ginseng on Depression Is Mediated by Lipids. Nutr. Neurosci..

[12] Wang C.-Z., Wu J.A., McEntee E., Yuan C.-S. (2006). Saponins Composition in American Ginseng Leaf and Berry Assayed by High-Performance Liquid Chromatography. J. Agric. Food Chem..

[13] Dong L., Xu J., Zhang L., Cheng R., Wei G., Su H., Yang J., Qian J., Xu R., Chen S. (2018). Rhizospheric Microbial Communities Are Driven by *Panax Ginseng* at Different Growth Stages and Biocontrol Bacteria Alleviates Replanting Mortality. Acta Pharm. Sin. B.

[14] Ahmed T., Raza S.H., Maryam A., Setzer W.N., Braidy N., Nabavi S.F., de Oliveira M.R., Nabavi S.M. (2016). Ginsenoside Rb1 as a Neuroprotective Agent: A Review. Brain Res. Bull..

[15] Zhou P., Xie W., He S., Sun Y., Meng X., Sun G., Sun X. (2019). Ginsenoside Rb1 as an Anti-Diabetic Agent and Its Underlying Mechanism Analysis. Cells.

[16] Zhou P., Xie W., Sun Y., Dai Z., Li G., Sun G., Sun X. (2019). Ginsenoside Rb1 and Mitochondria: A Short Review of the Literature. Mol. Cell. Probes.

[17] Kochan E., Kołodziej B., Gadomska G., Chmiel A. (2008). Ginsenoside Contents in *Panax Quinquefolium* Organs from Field Cultivation. Z. Für Nat. C.

[18] Sritularak B., Morinaga O., Yuan C.-S., Shoyama Y., Tanaka H. (2009). Quantitative Analysis of Ginsenosides Rb1, Rg1, and Re in American Ginseng Berry and Flower Samples by ELISA Using Monoclonal Antibodies. J. Nat. Med..

[19] Son T., Eguchi T., Shoyama Y., Tanaka H. (2019). ELISA for the Detection of Marker Compound for Crop Fertilizer Use of Various Medicinal Crop Extracts Using Bacterium. J.-Fac. Agric. Kyushu Univ..

[20] Miao L., Yang Y., Li Z., Fang Z., Zhang Y., Han C. (2022). Ginsenoside Rb2: A Review of Pharmacokinetics and Pharmacological Effects. J. Ginseng Res..

[21] Jin Y., Hao Y., Zhang H., Qu Z., Wang Y., Piao X. (2022). Dynamic Changes of Ginsenosides in *Panax Quinquefolium* Fruit at Different Development Stages Measured Using UHPLC-Orbitrap MS. Rapid Commun. Mass Spectrom..

[22] Li W., Duan Y., Yan X., Liu X., Fan M., Wang Z. (2022). A Mini-Review on Pharmacological Effects of Ginsenoside Rb3, a Marked Saponin from *Panax genus*. Biocell.

[23] Bae E.-A., Choo M.-K., Park E.-K., Park S.-Y., Shin H.-Y., Kim D.-H. (2002). Metabolism of Ginsenoside R_c_ by Human Intestinal Bacteria and Its Related Antiallergic Activity. Biol. Pharm. Bull..

[24] Kim D.H., Park C.H., Park D., Choi Y.J., Park M.H., Chung K.W., Kim S.R., Lee J.S., Chung H.Y. (2014). Ginsenoside Rc Modulates Akt/FoxO1 Pathways and Suppresses Oxidative Stress. Arch. Pharm. Res..

[25] Yu T., Yang Y., Kwak Y.-S., Song G.G., Kim M.-Y., Rhee M.H., Cho J.Y. (2017). Ginsenoside Rc from *Panax Ginseng* Exerts Anti-Inflammatory Activity by Targeting TANK-Binding Kinase 1/Interferon Regulatory Factor-3 and P38/ATF-2. J. Ginseng Res..

[26] Huang Q., Su H., Qi B., Wang Y., Yan K., Wang X., Li X., Zhao D. (2021). A SIRT1 Activator, Ginsenoside Rc, Promotes Energy Metabolism in Cardiomyocytes and Neurons. J. Am. Chem. Soc..

[27] Chen Y.-Y., Liu Q.-P., An P., Jia M., Luan X., Tang J.-Y., Zhang H. (2022). Ginsenoside Rd: A Promising Natural Neuroprotective Agent. Phytomedicine.

[28] Li Y., Wang M.-L., Zhang B., Fan X.-X., Tang Q., Yu X., Li L.-N., Fan A.-R., Chang H.-S., Zhang L.-Z. (2022). Antidepressant-Like Effect and Mechanism of Ginsenoside Rd on Rodent Models of Depression. Drug Des. Devel. Ther..

[29] Peng L., Sun S., Xie L.-H., Wicks S.M., Xie J.-T. (2012). Ginsenoside Re: Pharmacological Effects on Cardiovascular System. Cardiovasc. Ther..

[30] Madhi I., Kim J.-H., Shin J.E., Kim Y. (2021). Ginsenoside Re Exhibits Neuroprotective Effects by Inhibiting Neuroinflammation via CAMK/MAPK/NF-κB Signaling in Microglia. Mol. Med. Rep..

[31] Wang H., Lv J., Jiang N., Huang H., Wang Q., Liu X. (2021). Ginsenoside Re Protects against Chronic Restraint Stress-Induced Cognitive Deficits through Regulation of NLRP3 and Nrf2 Pathways in Mice. Phytother. Res..

[32] Lee B., Shim I., Lee H., Hahm D.-H. (2012). Effect of Ginsenoside Re on Depression- and Anxiety-like Behaviors and Cognition Memory Deficit Induced by Repeated Immobilization in Rats. J. Microbiol. Biotechnol..

[33] He F., Yu C., Liu T., Jia H. (2019). Ginsenoside Rg1 as an Effective Regulator of Mesenchymal Stem Cells. Front. Pharmacol..

[34] He F., Yao G. (2021). Ginsenoside Rg1 as a Potential Regulator of Hematopoietic Stem/Progenitor Cells. Stem Cells Int..

[35] Gao Y., Li J., Wang J., Li X., Li J., Chu S., Li L., Chen N., Zhang L. (2020). Ginsenoside Rg1 Prevent and Treat Inflammatory Diseases: A Review. Int. Immunopharmacol..

[36] Jiang B., Xiong Z., Yang J., Wang W., Wang Y., Hu Z.-L., Wang F., Chen J.-G. (2012). Antidepressant-like Effects of Ginsenoside Rg1 Are Due to Activation of the BDNF Signalling Pathway and Neurogenesis in the Hippocampus. Br. J. Pharmacol..

[37] Li X., Xiang N., Wang Z. (2020). Ginsenoside Rg2 Attenuates Myocardial Fibrosis and Improves Cardiac Function after Myocardial Infarction via AKT Signaling Pathway. Biosci. Biotechnol. Biochem..

[38] Liu G., Qi X., Li X., Sun F. (2021). Ginsenoside Rg2 Protects Cardiomyocytes against Trastuzumab-Induced Toxicity by Inducing Autophagy. Exp. Ther. Med..

[39] Wang Q., Fu W., Yu X., Xu H., Sui D., Wang Y. (2021). Ginsenoside Rg2 Alleviates Myocardial Fibrosis by Regulating TGF-Β1/Smad Signalling Pathway. Pharm. Biol..

[40] Gou D., Pei X., Wang J., Wang Y., Hu C., Song C., Cui S., Zhou Y. (2020). Antiarrhythmic Effects of Ginsenoside Rg2 on Calcium Chloride-Induced Arrhythmias without Oral Toxicity. J. Ginseng Res..

[41] Cui J., Shan R., Cao Y., Zhou Y., Liu C., Fan Y. (2021). Protective Effects of Ginsenoside Rg2 against Memory Impairment and Neuronal Death Induced by Aβ25-35 in Rats. J. Ethnopharmacol..

[42] Wang C.-Z., Zhang B., Song W.-X., Wang A., Ni M., Luo X., Aung H.H., Xie J.-T., Tong R., He T.-C. (2006). Steamed American Ginseng Berry: Ginsenoside Analyses and Anticancer Activities. J. Agric. Food Chem..

[43] Peng Z., Wu W.W., Yi P. (2020). The Efficacy of Ginsenoside Rg3 Combined with First-Line Chemotherapy in the Treatment of Advanced Non-Small Cell Lung Cancer in China: A Systematic Review and Meta-Analysis of Randomized Clinical Trials. Front. Pharmacol..

[44] Nakhjavani M., Smith E., Townsend A.R., Price T.J., Hardingham J.E. (2020). Anti-Angiogenic Properties of Ginsenoside Rg3. Molecules.

[45] Kim J., Shim J., Lee S., Cho W.-H., Hong E., Lee J.H., Han J.-S., Lee H.J., Lee K.W. (2016). Rg3-Enriched Ginseng Extract Ameliorates Scopolamine-Induced Learning Deficits in Mice. BMC Complement. Altern. Med..

[46] Tam D.N.H., Truong D.H., Nguyen T.T.H., Quynh L.N., Tran L., Nguyen H.D., Shamandy B.E., Le T.M.H., Tran D.K., Sayed D. (2018). Ginsenoside Rh1: A Systematic Review of Its Pharmacological Properties. Planta Med..

[47] Zhang H., Park S., Huang H., Kim E., Yi J., Choi S.-K., Ryoo Z., Kim M. (2021). Anticancer Effects and Potential Mechanisms of Ginsenoside Rh2 in Various Cancer Types (Review). Oncol. Rep..

[48] Lee D.-Y., Park C.W., Lee S.J., Park H.-R., Kim S.H., Son S.-U., Park J., Shin K.-S. (2019). Anti-Cancer Effects of *Panax Ginseng* Berry Polysaccharides via Activation of Immune-Related Cells. Front. Pharmacol..

[49] Wang C.-Z., Hou L., Wan J.-Y., Yao H., Yuan J., Zeng J., Park C.W., Kim S.H., Seo D.B., Shin K.-S. (2020). Ginseng Berry Polysaccharides on Inflammation-Associated Colon Cancer: Inhibiting T-Cell Differentiation, Promoting Apoptosis, and Enhancing the Effects of 5-Fluorouracil. J. Ginseng Res..

[50] Xie J.-T., Wang C.-Z., Zhang B., Mehendale S.R., Li X.-L., Sun S., Han A.H., Du W., He T.-C., Yuan C.-S. (2009). In Vitro and in Vivo Anticancer Effects of American Ginseng Berry: Exploring Representative Compounds. Biol. Pharm. Bull..

[51] Xie J.T., Wang C.Z., Ni M., Wu J.A., Mehendale S.R., Aung H.H., Foo A., Yuan C.S. (2007). American Ginseng Berry Juice Intake Reduces Blood Glucose and Body Weight in Ob/Ob Mice. J. Food Sci..

[52] Ko S.K., Bae H.M., Cho O.S., Im B.O., Chung S.H., Lee B.Y. (2008). Analysis of Ginsenoside Composition of Ginseng Berry and Seed. Food Sci. Biotechnol..

[53] Hou W., Wang Y., Zheng P., Cui R. (2020). Effects of Ginseng on Neurological Disorders. Front. Cell. Neurosci..

[54] Jin Y., Cui R., Zhao L., Fan J., Li B. (2019). Mechanisms of *Panax Ginseng* Action as an Antidepressant. Cell Prolif..

[55] Zhang H., Li Z., Zhou Z., Yang H., Zhong Z., Lou C. (2016). Antidepressant-like Effects of Ginsenosides: A Comparison of Ginsenoside Rb3 and Its Four Deglycosylated Derivatives, Rg3, Rh2, Compound K, and 20(S)-Protopanaxadiol in Mice Models of Despair. Pharmacol. Biochem. Behav..

[56] Liu M., Liu J., Zhang L., Geng Q., Ge Y. (2019). Antidepressant-like Effects of Ginseng Fruit Saponin in Myocardial Infarction Mice. Biomed. Pharmacother..

[57] He D.-F., Ren Y.-P., Liu M.-Y. (2016). Effects of Ginseng Fruit Saponins on Serotonin System in Sprague-Dawley Rats with Myocardial Infarction, Depression, and Myocardial Infarction Complicated with Depression. Chin. Med. J..

[58] Liu M.-Y., Ren Y.-P., Zhang L.-J., Ding J.Y. (2016). Pretreatment with Ginseng Fruit Saponins Affects Serotonin Expression in an Experimental Comorbidity Model of Myocardial Infarction and Depression. Aging Dis..

[59] Hu J.R., Chun Y.S., Kim J.K., Cho I.J., Ku S.K. (2019). Ginseng Berry Aqueous Extract Prevents Scopolamine-Induced Memory Impairment in Mice. Exp. Ther. Med..

[60] Shen H., Zhang Z., Jiang S., Jiang Z. (2006). Protective effects of ginsenoside RB3 on hypoxic/ischemic brain injury and involved mechanisms. Zhongguo Ying Yong Sheng Li Xue Za Zhi.

[61] Wang T., Yu X., Qu S., Xu H., Han B., Sui D. (2010). Effect of Ginsenoside Rb3 on Myocardial Injury and Heart Function Impairment Induced by Isoproterenol in Rats. Eur. J. Pharmacol..

[62] Sun J., Yu X., Huangpu H., Yao F. (2019). Ginsenoside Rb3 Protects Cardiomyocytes against Hypoxia/Reoxygenation Injury via Activating the Antioxidation Signaling Pathway of PERK/Nrf2/HMOX1. Biomed. Pharmacother..

[63] Bansal Y., Singh R., Parhar I., Kuhad A., Soga T. (2019). Quinolinic Acid and Nuclear Factor Erythroid 2-Related Factor 2 in Depression: Role in Neuroprogression. Front. Pharmacol..

[64] Jiang S., Fang D.-F., Chen Y. (2018). Involvement of N-Methyl-D-Aspartic Acid Receptor and DL-α-Amino-3-Hydroxy-5- Methyl-4-Isoxazole Propionic Acid Receptor in Ginsenosides Rb1 and Rb3 against Oxygen-Glucose Deprivation-Induced Injury in Hippocampal Slices from Rat. Pharmacology.

[65] Peng L.-L., Shen H.-M., Jiang Z.-L., Li X., Wang G.-H., Zhang Y.-F., Ke K.-F. (2009). Inhibition of NMDA Receptors Underlies the Neuroprotective Effect of Ginsenoside Rb3. Am. J. Chin. Med..

[66] Jiang S., Miao B., Song X., Jiang Z. (2011). Inactivation of GABA(A) Receptor Reduces Ginsenoside Rb3 Neuroprotection in Mouse Hippocampal Slices after Oxygen-Glucose Deprivation. J. Ethnopharmacol..

[67] Cui J., Jiang L., Xiang H. (2012). Ginsenoside Rb3 Exerts Antidepressant-like Effects in Several Animal Models. J. Psychopharmacol..

[68] Kim M.S., Yu J.M., Kim H.J., Kim H.B., Kim S.T., Jang S.K., Choi Y.W., Lee D.I., Joo S.S. (2014). Ginsenoside Re and Rd Enhance the Expression of Cholinergic Markers and Neuronal Differentiation in Neuro-2a Cells. Biol. Pharm. Bull..

[69] Kim D.H., Kim D.W., Jung B.H., Lee J.H., Lee H., Hwang G.S., Kang K.S., Lee J.W. (2019). Ginsenoside Rb2 Suppresses the Glutamate-Mediated Oxidative Stress and Neuronal Cell Death in HT22 Cells. J. Ginseng Res..

[70] Han S.-K., Joo M.-K., Kim J.-K., Jeung W., Kang H., Kim D.-H. (2020). Bifidobacteria-Fermented Red Ginseng and Its Constituents Ginsenoside Rd and Protopanaxatriol Alleviate Anxiety/Depression in Mice by the Amelioration of Gut Dysbiosis. Nutrients.

[71] Liu G.-M., Lu T.-C., Sun M.-L., Jia W.-Y., Ji X., Luo Y.-G. (2020). Ginsenoside Rd Inhibits Glioblastoma Cell Proliferation by Up-Regulating the Expression of MiR-144-5p. Biol. Pharm. Bull..

[72] Kim C., Spencer B., Rockenstein E., Yamakado H., Mante M., Adame A., Fields J.A., Masliah D., Iba M., Lee H.-J. (2018). Immunotherapy Targeting Toll-like Receptor 2 Alleviates Neurodegeneration in Models of Synucleinopathy by Modulating α-Synuclein Transmission and Neuroinflammation. Mol. Neurodegener..

[73] Wang X., Sundquist K., Hedelius A., Palmér K., Memon A.A., Sundquist J. (2015). Circulating MicroRNA-144-5p Is Associated with Depressive Disorders. Clin. Epigenetics.

[74] Sundquist K., Memon A.A., Palmér K., Sundquist J., Wang X. (2021). Inflammatory Proteins and MiRNA-144-5p in Patients with Depression, Anxiety, or Stress- and Adjustment Disorders after Psychological Treatment. Cytokine.

[75] Hyun T.K. (2021). A Recent Overview on Ginsenosides as MicroRNA Modulators in the Treatment of Human Diseases. EXCLI J..

[76] Toledo A.R.L., Monroy G.R., Salazar F.E., Lee J.-Y., Jain S., Yadav H., Borlongan C.V. (2022). Gut-Brain Axis as a Pathological and Therapeutic Target for Neurodegenerative Disorders. Int. J. Mol. Sci..

[77] Ortega M.A., Alvarez-Mon M.A., García-Montero C., Fraile-Martinez O., Guijarro L.G., Lahera G., Monserrat J., Valls P., Mora F., Rodríguez-Jiménez R. (2022). Gut Microbiota Metabolites in Major Depressive Disorder-Deep Insights into Their Pathophysiological Role and Potential Translational Applications. Metabolites.

[78] Ait Chait Y., Mottawea W., Tompkins T.A., Hammami R. (2021). Nutritional and Therapeutic Approaches for Protecting Human Gut Microbiota from Psychotropic Treatments. Prog. Neuro-Psychopharmacol. Biol. Psychiatry.

[79] Grootjans J., Thuijls G., Verdam F., Derikx J.P., Lenaerts K., Buurman W.A. (2010). Non-Invasive Assessment of Barrier Integrity and Function of the Human Gut. World J. Gastrointest. Surg..

[80] Rodrigues F.T.S., de Souza M.R.M., Lima C.N.d.C., da Silva F.E.R., Costa D.V.d.S., Dos Santos C.C., Miyajima F., de Sousa F.C.F., Vasconcelos S.M.M., Barichello T. (2018). Major Depression Model Induced by Repeated and Intermittent Lipopolysaccharide Administration: Long-Lasting Behavioral, Neuroimmune and Neuroprogressive Alterations. J. Psychiatr. Res..

[81] Grigoleit J.-S., Kullmann J.S., Wolf O.T., Hammes F., Wegner A., Jablonowski S., Engler H., Gizewski E., Oberbeck R., Schedlowski M. (2011). Dose-Dependent Effects of Endotoxin on Neurobehavioral Functions in Humans. PLoS ONE.

[82] Maes M., Kubera M., Leunis J.-C. (2008). The Gut-Brain Barrier in Major Depression: Intestinal Mucosal Dysfunction with an Increased Translocation of LPS from Gram Negative Enterobacteria (Leaky Gut) Plays a Role in the Inflammatory Pathophysiology of Depression. Neuro Endocrinol. Lett..

[83] Ren D.-D., Li S.-S., Lin H.-M., Xia Y.-S., Li Z.-M., Bo P.-P., Mu R., Zhao L.-J., Sun Y.-S. (2022). *Panax Quinquefolius* Polysaccharides Ameliorate Antibiotic-Associated Diarrhoea Induced by Lincomycin Hydrochloride in Rats via the MAPK Signaling Pathways. J. Immunol. Res..

[84] Li S., Huo X., Qi Y., Ren D., Li Z., Qu D., Sun Y. (2022). The Protective Effects of Ginseng Polysaccharides and Their Effective Subfraction against Dextran Sodium Sulfate-Induced Colitis. Foods.

[85] Fan J., Liu S., Ai Z., Chen Y., Wang Y., Li Y., Li X., Xiao S., Wang Y. (2021). Fermented Ginseng Attenuates Lipopolysaccharide-Induced Inflammatory Responses by Activating the TLR4/MAPK Signaling Pathway and Remediating Gut Barrier. Food Funct..

[86] Hu J., Yang J., Jiang S., Zhang J., Liu Z., Hou J., Gong X., Wang Y., Wang Z., Li W. (2021). *Panax Quinquefolium* Saponins Protect against Cisplatin Evoked Intestinal Injury via ROS-Mediated Multiple Mechanisms. Phytomedicine.

[87] Jeon H., Kim H.-Y., Bae C.-H., Lee Y., Kim S. (2020). Korean Red Ginseng Regulates Intestinal Tight Junction and Inflammation in the Colon of a Parkinson’s Disease Mouse Model. J. Med. Food.

[88] Wang K., Zhang H., Han Q., Lan J., Chen G., Cao G., Yang C. (2020). Effects of Astragalus and Ginseng Polysaccharides on Growth Performance, Immune Function and Intestinal Barrier in Weaned Piglets Challenged with Lipopolysaccharide. J. Anim. Physiol. Anim. Nutr..

[89] He L.-X., Wang J.-B., Sun B., Zhao J., Li L., Xu T., Li H., Sun J.-Q., Ren J., Liu R. (2017). Suppression of TNF-α and Free Radicals Reduces Systematic Inflammatory and Metabolic Disorders: Radioprotective Effects of Ginseng Oligopeptides on Intestinal Barrier Function and Antioxidant Defense. J. Nutr. Biochem..

[90] Zhou F., Zhang P., Chen X., Yan J., Yao J., Yu Z., Chen X. (2016). Ginsenoside Rb1 Protects the Intestinal Mucosal Barrier Following Peritoneal Air Exposure. Exp. Ther. Med..

[91] Lee E.-J., Song M.-J., Kwon H.-S., Ji G.E., Sung M.-K. (2012). Oral Administration of Fermented Red Ginseng Suppressed Ovalbumin-Induced Allergic Responses in Female BALB/c Mice. Phytomedicine.

[92] Seong M.A., Woo J.K., Kang J.-H., Jang Y.S., Choi S., Jang Y.S., Lee T.H., Jung K.H., Kang D.K., Hurh B.S. (2015). Oral Administration of Fermented Wild Ginseng Ameliorates DSS-Induced Acute Colitis by Inhibiting NF-ΚB Signaling and Protects Intestinal Epithelial Barrier. BMB Rep..

[93] Han K.-S., Balan P., Hong H.-D., Choi W.-I., Cho C.-W., Lee Y.-C., Moughan P.J., Singh H. (2014). Korean Ginseng Modulates the Ileal Microbiota and Mucin Gene Expression in the Growing Rat. Food Funct..

[94] Chen H., Yang H., Deng J., Fan D. (2021). Ginsenoside Rk3 Ameliorates Obesity-Induced Colitis by Regulating of Intestinal Flora and the TLR4/NF-ΚB Signaling Pathway in C57BL/6 Mice. J. Agric. Food Chem..

[95] Xia T., Zhang B., Li Y., Fang B., Zhu X., Xu B., Zhang J., Wang M., Fang J. (2020). New Insight into 20(S)-Ginsenoside Rh2 against T-Cell Acute Lymphoblastic Leukemia Associated with the Gut Microbiota and the Immune System. Eur. J. Med. Chem..

[96] Huang G., Khan I., Li X., Chen L., Leong W., Ho L.T., Hsiao W.L.W. (2017). Ginsenosides Rb3 and Rd Reduce Polyps Formation While Reinstate the Dysbiotic Gut Microbiota and the Intestinal Microenvironment in ApcMin/+ Mice. Sci. Rep..

[97] Qu L., Ma X., Fan D. (2021). Ginsenoside Rk3 Suppresses Hepatocellular Carcinoma Development through Targeting the Gut-Liver Axis. J. Agric. Food Chem..

[98] Bai X., Fu R., Duan Z., Wang P., Zhu C., Fan D. (2021). Ginsenoside Rk3 Alleviates Gut Microbiota Dysbiosis and Colonic Inflammation in Antibiotic-Treated Mice. Food Res. Int..

[99] Wei Y., Yang H., Zhu C., Deng J., Fan D. (2020). Hypoglycemic Effect of Ginsenoside Rg5 Mediated Partly by Modulating Gut Microbiota Dysbiosis in Diabetic Db/Db Mice. J. Agric. Food Chem..

[100] Xu Y., Wang N., Tan H.-Y., Li S., Zhang C., Feng Y. (2021). Gut-Liver Axis Modulation of *Panax Notoginseng* Saponins in Nonalcoholic Fatty Liver Disease. Hepatol. Int..

[101] Bai X., Fu R., Duan Z., Liu Y., Zhu C., Fan D. (2021). Ginsenoside Rh4 Alleviates Antibiotic-Induced Intestinal Inflammation by Regulating the TLR4-MyD88-MAPK Pathway and Gut Microbiota Composition. Food Funct..

[102] Zhou R., He D., Xie J., Zhou Q., Zeng H., Li H., Huang L. (2021). The Synergistic Effects of Polysaccharides and Ginsenosides from American Ginseng (*Panax Quinquefolius* L.) Ameliorating Cyclophosphamide-Induced Intestinal Immune Disorders and Gut Barrier Dysfunctions Based on Microbiome-Metabolomics Analysis. Front. Immunol..

[103] Long J., Liu X.-K., Kang Z.-P., Wang M.-X., Zhao H.-M., Huang J.-Q., Xiao Q.-P., Liu D.-Y., Zhong Y.-B. (2022). Ginsenoside Rg1 Ameliorated Experimental Colitis by Regulating the Balance of M1/M2 Macrophage Polarization and the Homeostasis of Intestinal Flora. Eur. J. Pharmacol..

[104] Liang W., Zhou K., Jian P., Chang Z., Zhang Q., Liu Y., Xiao S., Zhang L. (2021). Ginsenosides Improve Nonalcoholic Fatty Liver Disease via Integrated Regulation of Gut Microbiota, Inflammation and Energy Homeostasis. Front. Pharmacol..

[105] Sun Y., Chen S., Wei R., Xie X., Wang C., Fan S., Zhang X., Su J., Liu J., Jia W. (2018). Metabolome and Gut Microbiota Variation with Long-Term Intake of *Panax Ginseng* Extracts on Rats. Food Funct..

[106] Xu J., Li T., Xia X., Fu C., Wang X., Zhao Y. (2020). Dietary Ginsenoside T19 Supplementation Regulates Glucose and Lipid Metabolism via AMPK and PI3K Pathways and Its Effect on Intestinal Microbiota. J. Agric. Food Chem..

[107] Qu Q., Yang F., Zhao C., Liu X., Yang P., Li Z., Han L., Shi X. (2021). Effects of Fermented Ginseng on the Gut Microbiota and Immunity of Rats with Antibiotic-Associated Diarrhea. J. Ethnopharmacol..

[108] Han K.-H., Enomoto M., Pelpolage S., Nagata R., Fukuma N., Fukushima M. (2020). In Vitro Fermentation Potential of the Residue of Korean Red Ginseng Root in a Mixed Culture of Swine Faecal Bacteria. Food Funct..

[109] Fan J., Wang Y., You Y., Ai Z., Dai W., Piao C., Liu J., Wang Y. (2019). Fermented Ginseng Improved Alcohol Liver Injury in Association with Changes in the Gut Microbiota of Mice. Food Funct..

[110] Yang C.M., Han Q.J., Wang K.L., Xu Y.L., Lan J.H., Cao G.T. (2019). Astragalus and Ginseng Polysaccharides Improve Developmental, Intestinal Morphological, and Immune Functional Characters of Weaned Piglets. Front. Physiol..

[111] Qi Y.-L., Li S.-S., Qu D., Chen L.-X., Gong R.-Z., Gao K., Sun Y.-S. (2019). Effects of ginseng neutral polysaccharide on gut microbiota in antibiotic-associated diarrhea mice. Zhongguo Zhong Yao Za Zhi.

[112] Sun Y.-F., Zhang X., Wang X.-Y., Jia W. (2018). Effect of long-term intake of ginseng extracts on gut microbiota in rats. Zhongguo Zhong Yao Za Zhi.

[113] Li J., Li R., Li N., Zheng F., Dai Y., Ge Y., Yue H., Yu S. (2018). Mechanism of Antidiabetic and Synergistic Effects of Ginseng Polysaccharide and Ginsenoside Rb1 on Diabetic Rat Model. J. Pharm. Biomed. Anal..

[114] Shen H., Gao X.-J., Li T., Jing W.-H., Han B.-L., Jia Y.-M., Hu N., Yan Z.-X., Li S.-L., Yan R. (2018). Ginseng Polysaccharides Enhanced Ginsenoside Rb1 and Microbial Metabolites Exposure through Enhancing Intestinal Absorption and Affecting Gut Microbial Metabolism. J. Ethnopharmacol..

[115] Wang C.-Z., Huang W.-H., Zhang C.-F., Wan J.-Y., Wang Y., Yu C., Williams S., He T.-C., Du W., Musch M.W. (2018). Role of Intestinal Microbiome in American Ginseng-Mediated Colon Cancer Protection in High Fat Diet-Fed AOM/DSS Mice. Clin. Transl. Oncol..

[116] Wang D., Shao S., Zhang Y., Zhao D., Wang M. (2021). Insight Into Polysaccharides from *Panax ginseng* C. A. Meyer in Improving Intestinal Inflammation: Modulating Intestinal Microbiota and Autophagy. Front. Immunol..

[117] Zhou S.-S., Zhou J., Xu J.-D., Shen H., Kong M., Yip K.-M., Han Q.-B., Zhao Z.-Z., Xu J., Chen H.-B. (2021). Ginseng Ameliorates Exercise-Induced Fatigue Potentially by Regulating the Gut Microbiota. Food Funct..

[118] Zhang M., Wang Y., Wu Y., Li F., Han M., Dai Y., Zheng F., Yue H. (2021). In Vitro Transformation of Protopanaxadiol Saponins in Human Intestinal Flora and Its Effect on Intestinal Flora. Evid.-Based Complement. Altern. Med..

[119] Song M.-Y., Kim B.-S., Kim H. (2014). Influence of *Panax Ginseng* on Obesity and Gut Microbiota in Obese Middle-Aged Korean Women. J. Ginseng Res..

[120] Hong J.T., Lee M.-J., Yoon S.J., Shin S.P., Bang C.S., Baik G.H., Kim D.J., Youn G.S., Shin M.J., Ham Y.L. (2021). Effect of Korea Red Ginseng on Nonalcoholic Fatty Liver Disease: An Association of Gut Microbiota with Liver Function. J. Ginseng Res..

[121] Xu Y., Wang N., Tan H.-Y., Li S., Zhang C., Zhang Z., Feng Y. (2020). *Panax Notoginseng* Saponins Modulate the Gut Microbiota to Promote Thermogenesis and Beige Adipocyte Reconstruction via Leptin-Mediated AMPKα/STAT3 Signaling in Diet-Induced Obesity. Theranostics.

[122] Bai Y., Bao X., Mu Q., Fang X., Zhu R., Liu C., Mo F., Zhang D., Jiang G., Li P. (2021). Ginsenoside Rb1, Salvianolic Acid B and Their Combination Modulate Gut Microbiota and Improve Glucolipid Metabolism in High-Fat Diet Induced Obese Mice. PeerJ.

[123] Hou Z., Song F., Xing J., Zheng Z., Liu S., Liu Z. (2022). Comprehensive Fecal Metabolomics and Gut Microbiota for the Evaluation of the Mechanism of *Panax Ginseng* in the Treatment of Qi-Deficiency Liver Cancer. J. Ethnopharmacol..

[124] Zheng F., Zhang M.-Y., Wu Y.-X., Wang Y.-Z., Li F.-T., Han M.-X., Dai Y.-L., Yue H. (2021). Biotransformation of Ginsenosides (Rb1, Rb2, Rb3, Rc) in Human Intestinal Bacteria and Its Effect on Intestinal Flora. Chem. Biodivers..

[125] Ouyang J., Lin J., Isnard S., Fombuena B., Peng X., Marette A., Routy B., Messaoudene M., Chen Y., Routy J.-P. (2020). The Bacterium Akkermansia Muciniphila: A Sentinel for Gut Permeability and Its Relevance to HIV-Related Inflammation. Front. Immunol..

[126] Cheng D., Xie M.Z. (2021). A Review of a Potential and Promising Probiotic Candidate-Akkermansia Muciniphila. J. Appl. Microbiol..

[127] Mennigen R., Bruewer M. (2009). Effect of Probiotics on Intestinal Barrier Function. Ann. N. Y. Acad. Sci..

[128] Liu Q., Tian H., Kang Y., Tian Y., Li L., Kang X., Yang H., Wang Y., Tian J., Zhang F. (2021). Probiotics Alleviate Autoimmune Hepatitis in Mice through Modulation of Gut Microbiota and Intestinal Permeability. J. Nutr. Biochem..

[129] Uusitupa H.-M., Rasinkangas P., Lehtinen M.J., Mäkelä S.M., Airaksinen K., Anglenius H., Ouwehand A.C., Maukonen J. (2020). Bifidobacterium Animalis Subsp. Lactis 420 for Metabolic Health: Review of the Research. Nutrients.

[130] Pinto-Sanchez M.I., Hall G.B., Ghajar K., Nardelli A., Bolino C., Lau J.T., Martin F.-P., Cominetti O., Welsh C., Rieder A. (2017). Probiotic Bifidobacterium Longum NCC3001 Reduces Depression Scores and Alters Brain Activity: A Pilot Study in Patients with Irritable Bowel Syndrome. Gastroenterology.

[131] Yang Y., Zhao S., Yang X., Li W., Si J., Yang X. (2022). The Antidepressant Potential of Lactobacillus Casei in the Postpartum Depression Rat Model Mediated by the Microbiota-Gut-Brain Axis. Neurosci. Lett..

[132] Berni Canani R., Di Costanzo M., Leone L. (2012). The Epigenetic Effects of Butyrate: Potential Therapeutic Implications for Clinical Practice. Clin. Epigenetics.

[133] Chen G., Ran X., Li B., Li Y., He D., Huang B., Fu S., Liu J., Wang W. (2018). Sodium Butyrate Inhibits Inflammation and Maintains Epithelium Barrier Integrity in a TNBS-Induced Inflammatory Bowel Disease Mice Model. EBioMedicine.

[134] Ferreira T.M., Leonel A.J., Melo M.A., Santos R.R.G., Cara D.C., Cardoso V.N., Correia M.I.T.D., Alvarez-Leite J.I. (2012). Oral Supplementation of Butyrate Reduces Mucositis and Intestinal Permeability Associated with 5-Fluorouracil Administration. Lipids.

[135] Vanhoutvin S.A.L.W., Troost F.J., Hamer H.M., Lindsey P.J., Koek G.H., Jonkers D.M.A.E., Kodde A., Venema K., Brummer R.J.M. (2009). Butyrate-Induced Transcriptional Changes in Human Colonic Mucosa. PLoS ONE.

[136] Wang H.-B., Wang P.-Y., Wang X., Wan Y.-L., Liu Y.-C. (2012). Butyrate Enhances Intestinal Epithelial Barrier Function via Up-Regulation of Tight Junction Protein Claudin-1 Transcription. Dig. Dis. Sci..

[137] Zhang Q., Yun Y., An H., Zhao W., Ma T., Wang Z., Yang F. (2021). Gut Microbiome Composition Associated with Major Depressive Disorder and Sleep Quality. Front. Psychiatry.

[138] Kim D.-H. (2018). Gut Microbiota-Mediated Pharmacokinetics of Ginseng Saponins. J. Ginseng Res..

[139] Lee J., Lee E., Kim D., Lee J., Yoo J., Koh B. (2009). Studies on Absorption, Distribution and Metabolism of Ginseng in Humans after Oral Administration. J. Ethnopharmacol..

[140] Dong W.-W., Zhao J., Zhong F.-L., Zhu W.-J., Jiang J., Wu S., Yang D.-C., Li D., Quan L.-H. (2017). Biotransformation of *Panax Ginseng* Extract by Rat Intestinal Microflora: Identification and Quantification of Metabolites Using Liquid Chromatography-Tandem Mass Spectrometry. J. Ginseng Res..

[141] Chen W., Yao P., Vong C.T., Li X., Chen Z., Xiao J., Wang S., Wang Y. (2021). Ginseng: A Bibliometric Analysis of 40-Year Journey of Global Clinical Trials. J. Adv. Res..

[142] Choi H.S., Kim S., Kim M.J., Kim M.-S., Kim J., Park C.-W., Seo D., Shin S.S., Oh S.W. (2018). Efficacy and Safety of *Panax Ginseng* Berry Extract on Glycemic Control: A 12-Wk Randomized, Double-Blind, and Placebo-Controlled Clinical Trial. J. Ginseng Res..

[143] Choi Y.D., Park C.W., Jang J., Kim S.H., Jeon H.Y., Kim W.G., Lee S.J., Chung W.S. (2013). Effects of Korean Ginseng Berry Extract on Sexual Function in Men with Erectile Dysfunction: A Multicenter, Placebo-Controlled, Double-Blind Clinical Study. Int. J. Impot. Res..

